# An analytical coupled technique for solving nonlinear large-amplitude oscillation of a conservative system with inertia and static non-linearity

**DOI:** 10.1186/s40064-016-2089-5

**Published:** 2016-04-14

**Authors:** Md. Abdur Razzak, Md. Shamsul Alam

**Affiliations:** Department of Mathematics, Rajshahi University of Engineering and Technology (RUET), Kazla, Rajshahi, 6204 Bangladesh

**Keywords:** Homotopy perturbation method (HPM) and variational approach, Non-linear oscillation, Large amplitude, Cantilever beam

## Abstract

Based on a new trial function, an analytical coupled technique (a combination of homotopy perturbation method and variational method) is presented to obtain the approximate frequencies and the corresponding periodic solutions of the free vibration of a conservative oscillator having inertia and static non-linearities. In some of the previous articles, the first and second-order approximations have been determined by the same method of such nonlinear oscillator, but the trial functions have not been satisfied the initial conditions. It seemed to be a big shortcoming of those articles. The new trial function of this paper overcomes aforementioned limitation. The first-order approximation is mainly considered in this paper. The main advantage of this present paper is, the first-order approximation gives better result than other existing second-order harmonic balance methods. The present method is valid for large amplitudes of oscillation. The absolute relative error measures (first-order approximate frequency) in this paper is 0.00 % for large amplitude *A* = 1000, while the relative error gives two different second-order harmonic balance methods: 10.33 and 3.72 %. Thus the present method is suitable for solving the above-mentioned nonlinear oscillator.

## Background

Nonlinear oscillation problems are important in physical sciences, mechanical structures and engineering structures. Nonlinear vibration of oscillation systems are modeled by nonlinear differential equations. It is almost difficult to get exact solution for such nonlinear differential equations. Several methods have been used to solve weakly (small parameters, so-called perturbation parameters) nonlinear differential equations. Among all, most widely used technique is perturbation method (Marion 1970; Krylov and Bogoliubov 1947; Bogoliubov and Mitropolskii 1961; Nayfeh 1973; Nayfeh and Mook 1979; Nayfeh 1981). The perturbation method is not applied when a small parameter is absent in a nonlinear problem. Nonlinear of planar, large-amplitude free vibrations of a slender, inextensible cantilever beam carrying a lumped mass with rotary inertia at an intermediate position along its span is one of the problems that does not contain small parameter. In general, such problem is not always possible to get exact solution because of their complexity and thus the analytical approximate techniques must be needed to solve such problem. Moreover, there have been many strongly nonlinear problems arising in both science and engineering. To eliminate the limitations of classical perturbation technique, many analytical techniques such as variational iterative method (He et al. 2010; Herisanu and Marinca 2010a, b), variational method (He 2007; Kaya et al. 2010; Khan et al. 2011), energy balance method (EBM) (He 2002, 2006), homotopy analysis method (Liao 2003) used to solve strongly nonlinear problems. Recently, Khan et al. (2013) generalized the standard homotopy analysis method to solve nonlinear oscillators with rational terms. Moreover, Khan and Mirzabeigy (2014) has been improved He’s energy balance method, especially the second-order approximation is considered here.

The homotopy perturbation method (HPM) (He 1999, 2004; Rafei et al. 2007; Ganji and Sadighi 2006; Ghorbani and Nadjafi 2007) is another effective technique for solving strongly nonlinear problems. The homotopy perturbation method was first introduced by He (1999). Generally, it is a method which is a combination of the classical perturbation method and the homotopy method in topology (He 2000). The solution procedure of HPM is very simple, only a few iteration steps lead to accurate approximations. Recently, some authors (Wang et al. 2012; Khan et al. 2014; Aminikhaha and Hemmatnezhad 2011; Akindeinde 2015; Suleman and Wu 2015) have been improved and modified the homotopy perturbation method. Moreover, another modified version of HPM named as optimal homotopy perturbation method (OHPM) (Marinca and Herisanu 2010, 2011; Herisanu and Marinca 2012) have also been used for solving strongly nonlinear systems. Furthermore, some authors (Akbarzade 2010; Khan et al. 2012; Akbarzade and Khan 2012) have developed an analytical approximate technique coupling of the homotopy perturbation method and variational method in order to get high accuracy. Khan et al. (2012) obtained fourth-order approximations of strongly nonlinear problems, but the solution procedures of third and fourth-order approximations are very laborious process. On the contrary, some authors (Akbarzade and Khan 2012; Hamdan and Dado 1997; Wu et al. 2003; Herisanu and Marinca 2010a, b) have investigated the free vibration of a conservative oscillator having inertia and static non-linearity. In the previous articles (Hamdan and Dado 1997; Wu et al. 2003), the second-order approximate frequencies as well as the corresponding periodic solutions of such nonlinear oscillator were determined by using harmonic balance method. The results of Hamdan and Dado (1997) and Wu et al. (2003) are valid for weak nonlinearities and small amplitudes of oscillation. Moreover, in terms of large amplitudes, the results of the researchers (Hamdan and Dado (1997) and Wu et al. (2003) do not provide better outcome. On the other hand, the solution procedure of the article (Herisanu and Marinca 2010a, b) is very laborious. One of the shortcomings of the articles (Akbarzade 2010; Akbarzade and Khan 2012) is their trial functions do not satisfy the initial conditions.

In this paper, an analytical coupled method [a combination of homotopy perturbation method (He 2004) and variational method (He 2007)], along with a new trial function, has been presented to obtain the approximate frequency and the corresponding periodic solution of the strongly nonlinear oscillation of a conservative oscillator having inertia and static non-linearities (Akbarzade and Khan 2012; Hamdan and Dado 1997; Wu et al. 2003; Herisanu and Marinca 2010a, b). The new trial function of the present paper has satisfied the initial conditions. The results obtained in this paper (first-order approximate frequencies) are much better result for large values of amplitude than other existing results (Hamdan and Dado 1997; Wu et al. 2003). The method is very easy and straightforward.

## Formulation and solution method

Consider the nonlinear oscillator (Hamdan and Dado 1997; Wu et al. 2003; Herisanu and Marinca 2010a, b)

1 $$\frac{{d^{2} u}}{{dt^{2} }} + \,u + \alpha \,u^{2} \frac{{d^{2} u}}{{dt^{2} }} + \alpha \,u\left( {\frac{du}{dt}} \right)^{2} + \beta \,u^{3} = 0,$$

subject to the initial conditions

2 $$u(0) = A,\quad \frac{du}{dt}(0) = 0.$$

By considering the nonlinear oscillator, Eq. (1), the following homotopy can be constructed:

3 $$u^{\prime \prime } + \omega^{2} u + p\left[ {\alpha \,u^{2} u^{\prime \prime } + \alpha \,uu^{\prime 2} + \beta \,u^{3} + (1 - \omega^{2} )u} \right] = 0,$$

where $p \in \,[0,1]$ and $\omega$ is an unknown angular frequency of the nonlinear oscillator which is further to be determined. When $p = 0$, Eq. (3) becomes the linearized equation, $u^{\prime\prime} + \omega^{2} u = 0.$ When $p = 1$, it turns out to be the original one.

Let us consider that the periodic solution to Eq. (1) may be written as a power series in *p*:

4 $$u = u_{0} + pu_{1} + p^{2} u_{2} + \cdots .$$

Substituting Eq. (4) into Eq. (3) and equating the coefficients of $p^{0}$ and $p^{1}$, we obtain

5 $$u^{\prime\prime}_{0} + \omega^{2} u_{0} = 0,\quad u_{0} (0) = A,\quad u^{\prime}_{0} (0) = 0,$$

and

6 $$u_{1}^{\prime \prime } + \omega^{2} u_{1} + \alpha \,u_{0}^{2} u_{0}^{\prime \prime } + \alpha \,u_{0} u_{0}^{\prime 2} + \beta \,u_{0}^{3} + (1 - \omega^{2} )u_{0} = 0,\quad u_{1} (0) = 0,\quad u_{1}^{\prime } (0) = 0.$$

The solution of Eq. (5) is $u_{0} = A\cos \omega \,t$, where $\omega$ will be determined from the variational formulation for $u_{1}$, which reads:

7 $$\begin{aligned} J(u_{1} ) & = \int\limits_{0}^{T} {\left\{ { - \frac{1}{2}u_{1}^{\prime 2} u_{1}^{\prime \prime } + \frac{1}{2}\omega^{2} u_{1}^{2} + \alpha \,u_{0}^{2} u_{0}^{\prime \prime } u_{1} + \alpha \,u_{0} u_{0}^{\prime 2} u_{1} + \beta \,u_{0}^{3} u_{1} + (1 - \omega^{2} )u_{0} u_{1} } \right\}} dt, \\ &\quad T = \frac{2\pi }{\omega }. \end{aligned}$$

In previous article (Akbarzade and Khan 2012), a trial function was chosen in the following form:

8 $$u_{1} (t) = B\left( {\cos \omega \,t - \frac{1}{3}\cos 5\omega \,t} \right)$$

The accuracy of the first-order approximate solution, Akbarzade and Khan (2012) was chosen the trial function in the following form:

9 $$u_{1} (t) = B_{1} \left( {\cos \omega \,t - \frac{1}{3}\cos 3\omega \,t} \right) + B_{3} \left( {\frac{1}{3}\cos 3\omega \,t - \frac{3}{5}\cos 5\omega \,t + \frac{5}{7}\cos 7\omega \,t} \right).$$

Here, we observe that the trial functions Eqs. (8)–(9) are not satisfied the initial conditions $u_{1} (0) = 0,\;u^{\prime}_{1} (0) = 0$ when substitutes $t = 0$ in the Eqs. (8)–(9). It is the main shortcomings of the article Akbarzade and Khan (2012).

In this paper, the limitation of the article Akbarzade and Khan (2012) has been removed by choosing a simple new trial function in the following form:

10 $$u_{1} (t) = B\left( {\cos \omega \,t - \frac{1}{3}\cos 3\omega \,t - \frac{2}{3}\cos 5\omega \,t} \right).$$

The new trial function given in Eq. (10) is satisfied the initial conditions $u_{1} (0) = 0,\;u^{\prime}_{1} (0) = 0.$ The trial function given in Eq. (10) makes the solution rapidly converges; furthermore, the determination of first-order approximation is very easy.

Substituting $u_{1}$ into functional Eq. (7), we obtain the following result:

11 $$J(A,B,\omega ) = \frac{{B\pi \left( {9A + 6A^{3} \beta - (9A + 52B + 3\,\alpha \,A^{3} )\omega^{2} } \right)}}{9\omega }.$$

Setting:

12 $$\frac{\partial J}{\partial B} = 0,\quad \frac{\partial J}{\partial \omega } = 0.$$

Solving Eq. (12), we obtain the first approximate frequency as a function of amplitude as

13 $$\omega = \omega_{0} = \sqrt {\frac{{3 + 2A^{2} \beta }}{{3 + \alpha \,A^{2} }}} ,$$

where $\omega_{0}$ is the first-order analytical approximate frequency.

Therefore, the first-order approximate solution of Eq. (1) becomes

14 $$u(t) = A\cos \omega \,t,$$

where $\omega$ is given in Eq. (13).

Thus, the determination of first-order approximation is very easy and straightforward. On the other hand, the determination of second-order approximation of the article (Herisanu and Marinca 2010a, b) is very laborious process; thus, seems to be complex.

## Results and discussion

An analytical coupled technique [combining of the homotopy perturbation method (He 2004) and variational method (He 2007)], along with a simple new trial function, has been presented to determine the approximate frequency and the corresponding periodic solution of the above-mentioned nonlinear oscillator given by Eq. (1). Recently, some authors (Akbarzade and Khan 2012; Hamdan and Dado 1997; Wu et al. 2003; Herisanu and Marinca 2010a, b) have determined the approximate frequencies and the periodic solutions of such nonlinear oscillator. They (Akbarzade and Khan 2012; Hamdan and Dado 1997; Wu et al. 2003; Herisanu and Marinca 2010a, b) were obtained second-order approximation because their first-order approximation did not provide better result. On the other hand, the solution procedures of the article (Herisanu and Marinca 2010a, b) are not easy and it is very laborious process also. In this situation, the first-order approximation of the present paper gives significantly better result than other existing second-order approximations (Hamdan and Dado 1997; Wu et al. 2003).

To verify the efficiency and accuracy of the present method, the approximate frequencies have been obtained for several amplitudes when $\alpha = \beta = 1$ and $\alpha = \beta = 2$ and have been compared those results with other existing harmonic balance methods (Hamdan and Dado 1997; Wu et al. 2003). All results are shown respectively in Tables 1 and 2. The absolute relative errors of the present paper (first-order frequencies) have been compared with the numerical frequency and give less than 0.00 % in the limit as $A \to \infty$ whereas the absolute relative errors of the first and second-order analytical approximations (obtained by Hamdan and Dado 1997) give less than 13.40 and 10.33 %, respectively. On the other hand, the absolute relative errors of the first and second-order analytical approximations (obtained by Wu et al. 2003) give less than 13.40 and 3.72 %, respectively. Thus, the convergent rate of the present method is very faster than Hamdan and Dado (1997); Wu et al. (2003).

**Table 1:** Comparison between the numerical frequency $\omega$, the approximate frequency obtained by present method (given in Eq. 13) and other existing frequencies (Hamdan and Dado 1997; Wu et al. 2003) for $\alpha = \beta = 1$ as well as several large amplitudes

*A*	Numerical frequency $\omega_{e}$	Hamdan and Dado (1997) (error%)		Wu et al. (2003) (error%)		Present method (error%) $\omega_{0}$
		$\omega_{0}$	$\omega_{1}$	$\omega_{0}$	$\omega_{1}$	
5	1.34288	1.20953	1.24841	1.20953	1.32217	1.37581
		9.93	7.04	9.93	1.54	2.45
10	1.38928	1.22074	1.26285	1.22074	1.35084	1.40388
		12.13	9.10	12.13	2.77	1.05
15	1.40138	1.22295	1.26570	1.22295	1.35672	1.40955
		12.73	9.68	12.73	3.19	0.58
20	1.40632	1.22373	1.26671	1.22373	1.35883	1.41158
		12.98	9.92	12.98	3.38	0.38
25	1.40883	1.22409	1.26718	1.22409	1.35981	1.41252
		13.11	10.05	13.11	3.48	0.26
30	1.41029	1.22429	1.26743	1.22429	1.36035	1.41304
		13.19	10.13	13.19	3.54	0.20
50	1.41261	1.22458	1.26781	1.22458	1.36113	1.41379
		13.31	10.25	13.31	3.65	0.08
100	1.41375	1.22470	1.26797	1.22470	1.36147	1.41411
		13.37	10.31	13.37	3.70	0.03
200	1.41408	1.22473	1.26801	1.22473	1.36155	1.41419
		13.39	10.33	13.39	3.72	0.00
500	1.41419	1.22474	1.26802	1.22474	1.36157	1.41421
		13.40	10.33	13.40	3.72	0.00
1000	1.41421	1.22474	1.26802	1.22474	1.36157	1.41421
		13.40	10.33	13.40	3.72	0.00

The absolute relative error has been also computed

**Table 2:** Comparison between the numerical frequency $\omega$, the approximate frequency obtained by present method (given in Eq. 13) and other existing frequency (Hamdan and Dado 1997; Wu et al. 2003) for $\alpha = \beta = 2$ as well as several large amplitudes

*A*	Numerical frequency $\omega_{e}$	Hamdan and Dado (1997) (error%)		Wu et al. (2003) (error%)		Present method (error%) $\omega_{0}$
		$\omega_{0}$	$\omega_{1}$	$\omega_{0}$	$\omega_{1}$	
5	1.37132	1.21687	1.25786	1.21687	1.34073	1.39406
		11.26	8.27	11.26	2.23	1.66
10	1.40006	1.22272	1.26541	1.22272	1.35613	1.40898
		12.67	9.61	12.67	3.14	0.64
15	1.40707	1.22384	1.26685	1.22384	1.35913	1.41187
		13.02	9.96	13.02	3.41	0.34
20	1.40986	1.22424	1.26736	1.22424	1.36020	1.41289
		13.17	10.11	13.17	3.52	0.22
25	1.41127	1.22442	1.26760	1.22442	1.36069	1.41337
		13.24	10.18	13.24	3.59	0.15
30	1.41207	1.22452	1.26773	1.22452	1.36096	1.41363
		13.28	10.22	13.28	3.62	0.11
50	1.41335	1.22466	1.26791	1.22466	1.36135	1.41400
		13.35	10.29	13.35	3.68	0.05
100	1.41397	1.22472	1.26799	1.22472	1.36152	1.41416
		13.39	10.32	13.39	3.71	0.01
200	1.41414	1.22474	1.26801	1.22474	1.36156	1.41420
		13.40	10.33	13.40	3.72	0.00
500	1.41420	1.22474	1.26802	1.22474	1.36157	1.41421
		13.40	10.33	13.40	3.72	0.00
1000	1.41421	1.22474	1.26802	1.22474	1.36158	1.41421
		13.40	10.34	13.40	3.72	0.00

The absolute relative error has been also computed

**Fig. 1:**
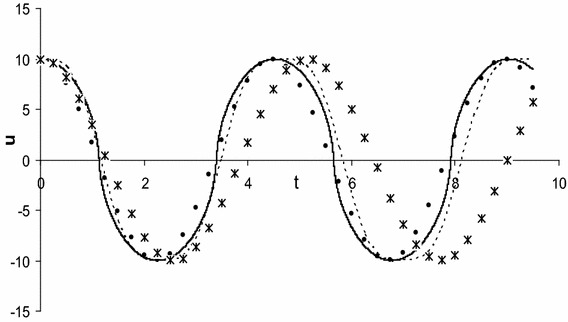
Comparison of the analytical approximate periodic solution obtained by present method (denoting by *circles line*) with numerical solution obtained by fourth order Runge–Kutta method (denoted by *solid line*) and also with the first-order (denoted by *cross lines*) as well as second-order (denoted by *dash line*) approximations obtained by harmonic balance method (Wu et al. 2003) for $\alpha = \beta = 1,\,\,A = 10$

**Fig. 2:**
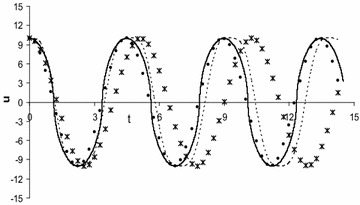
Comparison of the analytical approximate periodic solution obtained by present method (denoting by *circles line*) with numerical solution obtained by fourth order Runge–Kutta method (denoted by *solid line*) and also with the first-order (denoted by *cross lines*) as well as second-order (denoted by *dash line*) approximations obtained by harmonic balance method (Wu et al. 2003) for $\alpha = \beta = 2,\,\,A = 10$

Next, the approximate solution of Eq. (1) has been determined by using present method and harmonic balance method (Wu et al. 2003) for $\alpha = \beta = 1,\,\,A = 10$ and shown in Fig. 1. Finally, the approximate solution of Eq. (1) has been determined by using present method and harmonic balance method (Wu et al. 2003) for $\alpha = \beta = 2,\,\,A = 10$ and shown in Fig. 2. All figures include numerical solution obtained by fourth order Runge–Kutta method.

From all the figures, we see that the first-order approximate solution obtained by harmonic balance method deviates from numerical solution. Moreover, the second-order approximate solution obtained by harmonic balance method does not better agreement with the corresponding numerical solution. On the other hand, the first-order approximate solution obtained by present method gives excellent agreement with the corresponding numerical solution. Therefore, the present method is suitable for solving Eq. (1) than Akbarzade and Khan (2012); Hamdan and Dado (1997); Wu et al. (2003); Herisanu and Marinca (2010a, b) for strong nonlinearity as well as large amplitudes of oscillation.

## Conclusion

In this paper, a simple analytical technique has been presented to solve of nonlinear oscillations of planar, flexural large amplitudes free vibration of a slender, inextensible cantilever beam carrying a lumped mass with rotary inertia at an intermediate position along its span. Generally, the first-order approximation is considered in this paper. The first-order approximation gives rapidly converges to the corresponding numerical solution. The present method gives better result than other existing results for large amplitudes of oscillation. It has been proved that the present method is very effective and convenient and provides more accurate result for solving strongly nonlinear oscillators.
